# Gait Generation Method of Snake Robot Based on Main Characteristic Curve Fitting

**DOI:** 10.3390/biomimetics8010105

**Published:** 2023-03-06

**Authors:** Chaoquan Tang, Lulu Sun, Gongbo Zhou, Xin Shu, Hongwei Tang, Hao Wu

**Affiliations:** Jiangsu Key Laboratory of Mine Mechanical and Electrical Equipment, School of Mechatronic Engineering, China University of Mining and Technology, Xuzhou 221116, China

**Keywords:** snake robot, backbone curve, gait generation method, snake robot control

## Abstract

Gait generation method is one of the important contents of snake robot motion control. Different gait generation methods produce completely different forms of control functions, so snake robots need more complicated programming logic and processes to realize various gaits and their transformation. Therefore, we propose a new unified expression of gait method, The MCC (main characteristics control) method simplifies and unifies the control functions of different snake robots gaits by extracting the main features of the backbone curves of snake robots gaits. Since all periodic curves that meet the Dirichlet conditions can be formed by superposition of sinusoidal curves, taking the “lowest frequency” part that reflects the main characteristics of the curve as the target configuration can simplify the motion control function of snake robots’ gaits. Based on the MCC method, some snake robot gaits are reconstructed, including serpentine gait, rolling gait, helix rolling gait, and crawler gait. In addition, based on MCC method, an AEH-sidewinding gait control method is proposed. The backbone of the AEH-sidewinding gait is closer to the ideal elliptic helix, thus improving the accuracy of its kinematics modeling of snake robot sidewinding gait. Finally, the validity of this gait is verified by experiments. This unified gait expression of snake robots will be helpful to realize smooth gait switching between different gaits of snake robots.

## 1. Introduction

With super redundant degrees of freedom, snake robots have been deployed in various scenarios, including search and rescue and archaeology [1,2]. The robot is also used in biology and bionic engineering research [3].

Snake robot completes complex tasks through gait movement. Tesch classified snake robot motion control gaits into two categories: parameterized and scripted [4]. The parameterized gait can directly define the joint angle during the motion of the snake robot through a set of parameterized equations. We can use several parameters with clear physical meaning to control the motion configuration of snake robot, such as lateral undulatory and rolling [5]. However, when the target configuration of snake robot becomes complex, it is difficult to directly define the corresponding joint angle, so scripted gait is produced. Different gait configurations can be generated by changing the input parameters, which makes the snake robot have the ability to carry out complex motion in multiple environments, so it has been widely used [6,7].

Without using gait functions, scripted gaits are often implemented based on backbone. In this method, the snake robot configuration is approximated as a continuous backbone curve [8,9]. Yamada mathematically modeled the target curve based on the *Frenet-Serret* reference frame, decomposed the curvature into the corresponding axis directions of the yaw and pitch joints on the backbone, and deduced the rotation angles of each joint of the snake robot [10]. Furthermore, adaptation to the environment is achieved by combining torque feedback with motion planning using this approximation method [11,12]. When the curvature and torsion of the target curve are known, it is easy to obtain the joint angle of the snake robot.

The key to using Yamada’s approximation method is to design and analyze the target curve. The most direct method is to design the three-dimensional coordinate function and then calculate the curvature and torsion and finally to obtain each joint angle of the snake robot. Zhen et al. proposed a rolling hump, whose target configuration is a curve obtained by superimposing a hump and an arc curve, which can make the snake robot climb over the ground obstacles [13]. Takemori also creatively proposed a design method of a target curve based on a connecting curve, including straight lines, circular arcs, helix arcs, and any curve with known curvature and torsion [14]. Based on this method, Takemori proposed the crawler gait and C-pedal gait [15]. When the snake robot adopts the crawler gait motion, it resembles a moving crawler, so it shows good adaptability to most terrains. The gait generation method of the connecting curve greatly reduces the difficulty of gait design and enriches the gait types of snake robots.

For this research situation, various gait control functions can be obtained by different generation methods. However, for some complex curves, the process of solving curvature and deflection through coordinates is complicated, and the resulting equation is not easy to express [16]. In addition, if there is a part with zero curvature in the target curve, the torsion may tend to infinity. Although the method based on connecting curve solves the problem, it leads to a new problem, which makes the control code of the snake robot have multiple judgment processes, and the control degree becomes more complicated

To address these issues, we propose a new gait generation method, the MCC (main characteristics curve) method, to generate control function for snake robots. This method is based on the assumption that any gait of a snake robot is periodic. We extract the main features of the periodic curve and take the “lowest frequency” part as the gait target configuration curve. Based on this idea, the main characteristic curve (MCC) method is proposed. The gait control equations designed based on the MCC method has unified expression, and the control code of snake robots can be simplified.

The MCC method is described in Section 2. In Section 3, based on the MCC method, we realize some typical snake robot gaits, including serpentine gait, rolling gait, helix rolling gait, and crawler gait. In Section 4, the AEH-sidewinding gait is designed, whose backbone curve will be closer to the ideal assumption of kinematics modeling. 

## 2. The MCC (Main Characteristic Curve) Method

**Figure 1:**
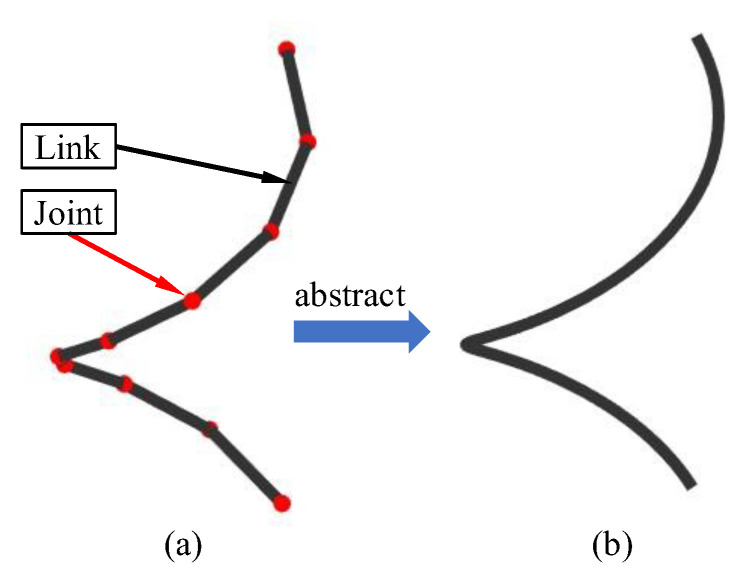
The abstractgou process from gait motion configuration of snake robot to backbone curve. (**a**) Snake robot configuration. (**b**) backbone curve of snake robot.

Since all periodic curves that meet the Dirichlet conditions can be formed by superposition of sinusoidal curves, taking the “lowest frequency” part that reflects the main characteristics of the curve as the target configuration can simplify the motion control function of snake robots’ gaits. The MCC method needs a different abstract backbone curve of snake robot gaits and intercepts its low frequency part, thus forming the control function of the snake robots. The backbone curve, which can be understood as an abstract curve that retains the basic configuration information of the robot, is to better represent the configuration characteristics of the robot, and it was introduced by Burdick et al. [3]. Figure 1 shows the difference between the spatial configuration of the snake robot and the abstract backbone curve. When designing the gait of snake robot, we can focus on the shape change in backbone. First, the gait target configuration curve is designed, and then the snake robot’s backbone is used to fit the curve and cycle so as to achieve the purpose of gait movement.

When the curvature κ(s) and torsion τ(s) of the target curve are known, [10] proposed a method to fit the backbone curve of the snake robot to the target configuration curve so as to obtain the joint angle of the snake robot. The principle is easy to understand, and the process is easy to realize. This process is briefly described below.

**Figure 2:**
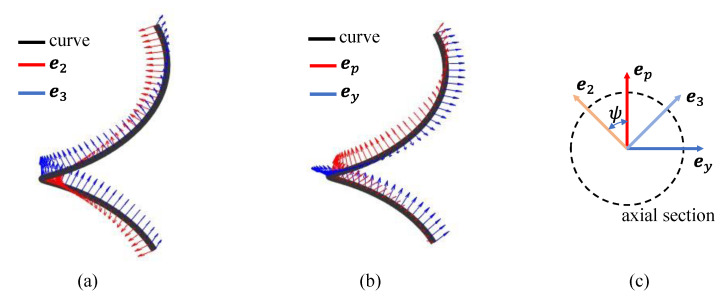
Difference between the Frenet–Serret frame and backbone curve reference set. (**a**) Direction diagram of curvature and deflection on the Frenet-Serret frame. (**b**) The component of curvature on the backbone curve reference set after the torsion direction. (**c**) The relationship between the Frenet-Serret frame and backbone curve reference set at axial section.

The method in [10] is based on the Frenet-Serret reference system. Let the backbone curve of the snake robot be ***c***(*s*), where s is the length variable along the curve. In Figure 2a, ***e***_1_(*s*), ***e***_2_(*s*), and ***e***_3_(*s*) are the unit vectors forming the standard orthogonal basis, which is called the Frenet-Serret reference system. The curvature *κ*(*s*) on the target curve corresponds to the change of ***e***_1_(*s*), and the torsion *τ*(*s*) corresponds to the change of ***e***_2_(*s*). Compared with the Frenet-Serret model, the orientation of the backbone curve needs to be considered in modeling.

Considering the backbone of snake robot as a continuous curve. Based on this definition, three basis vectors of the backbone reference frame are ***e****_r_*(*s*), ***e****_p_*(*s*), and ***e****_y_*(*s*). ***e****_r_*(*s*) is equal to ***e***_1_(*s*), ***e****_p_*(*s*) is the unit vector along the yaw axis at the curve ***c***(*s*), and ***e****_y_*(*s*) is the unit vector along the pitch axis at the curve ***c***(*s*), as shows in Figure 2b. These vectors form the basis vectors of the backbone curve reference frame. *ψ*(*s*) is the twist angle of the Frenet-Serret reference frame relative to the backbone curve reference frame along ***e****_r_*(*s*), as shown in Figure 2. Based on this method, the *ψ*(*s*) can be obtained by integrating over:

(1) $$\psi (s)=\int_{0}^{s}\tau (s)ds+\psi (0)$$

where *ψ*(0) is an arbitrary integral constant corresponding to the initial angle. By changing the value, an action of rolling along the axis can be generated.

$\kappa_{p}(s)$ and $\kappa_{y}(s)$ are the curvature of the backbone curve in the direction of the pitch axis and the yaw axis, respectively, which can be obtained:

(2) $$\kappa_{p}(s)=-\kappa \cdot \sin(\psi (s)),\;\kappa_{y}(s)=\kappa \cdot \cos(\psi (s))$$

Finally, the angle of each joint of the snake robot can be obtained:

(3) $$\left\{\begin{array}{cc} \theta_{p}(s)=\int_{s-L_{0}}^{s+L_{0}}k_{p}(\hat{s})d\hat{s} & \left(odd\right)\\ \theta_{y}(s)=\int_{s-L_{0}}^{s+L_{0}}k_{y}(\hat{s})d\hat{s} & \left(even\right) \end{array}\right.$$

where $L_{0}$ is the distance between adjacent joint centers of orthogonal snake robot, $\theta_{p}(s)$ is the joint angle of the yaw joint at the *s* on the snake robot backbone curve, and $\theta_{y}(s)$ is the joint angle of the pitch joint at the *s* point on the snake robot backbone curve.

The difficulty of above method lies in the design and analysis of the target configuration curve. It is difficult to analytically represent complex target forms of snake robots. Using the method proposed in [12] can avoid the problem that the torsion tends to infinity, but the obtained target curve configuration analytic function is complicated. Therefore, it is necessary to come up with a method that can obtain the target curve that is easy to analyze, as well as to avoid the situation that the curvature, κ(s), or torsion, κ(s), and torsion, τ(s), is infinite.

Consider that the backbone of the snake robot is a periodic curve of finite length when performing gait motion. For any periodic curve, as long as certain conditions are met, it can be understood as the superposition of infinite sinusoids, of which the “lowest frequency” part reflects the main characteristics of the curve.

Therefore, based on the main characteristics of the snake robot target configuration curve, the general expression of the snake robot target configuration curve, namely, the main characteristic curve (MCC) method, is proposed:

(4) $$\left\{\begin{array}{c} \kappa (s)=A_{1}+B_{1}\cdot \sin(\omega_{1}s+\varphi_{1})\\ \tau (s)=A_{2}+B_{2}\cdot \sin(\omega_{2}s+\varphi_{2}) \end{array}\right.$$

## 3. Gait Analysis Based on the MCC Method

Based on the MCC method, the common gaits of snake robots, including parameterized gaits and scripted gaits, are analyzed to verify the versatility and reliability of the proposed expression.

### 3.1. Some Example Gaits for Parameterized Gaits 

Parametric gait refers to a gait that can describe the change in joint angle during robot motion by a simple equation [1]. In the field of snake robots gait research, parametric gaits are generated based on sine waves in horizontal and vertical planes. Therefore, when the snake robot adopts parametric gait, its joint angle can be expressed as:

(5) $$\left\{\begin{array}{l} \theta_{p}(s,t)=\theta_{p,con}+\theta_{p,A}\sin(\phi (s,t))\\ \theta_{y}(s,t)=\theta_{y,con}+\theta_{y,A}\sin(\phi (s,t)+\delta ) \end{array}\right.$$

where $\theta_{con}$, $\theta_{A}$, ϕ, δ are, respectively, offset, amplitude, frequency, and phase shift terms of the pitch and yaw joints. 

#### 3.1.1. Serpentine Gait (*A*_2_ = 0, *B*_2_ = 0)

Serpentine motion is the most typical parametric gait of the snake robot, which was first proposed by Hirose. Let *A*_2_ = 0, *B*_2_ = 0, and we can obtain the target configuration curve (two-dimensional plane curve) of the snake robot’s serpentine gait: $\kappa (s)=A_{1}+B_{1}\sin(\omega_{1}s+\varphi_{1})$. At this time, although the curvature changes periodically, the target configuration curve of the snake shaped robot changes from a three-dimensional space curve to a two-dimensional plane curve because the torsion is constant to 0. By changing the value of $A_{1}$, $B_{1}$, $\omega_{1}$, we can obtain the configuration curves of snake-like robots with different shapes. Specifically, when $A_{1}=0$,$B_{1}=ab$(a≠0, b≠0), we obtain the *Serpenoid* curve: κ(s)=αbsin(bs), τ(s)=0. Where a is the angle between *serpenoid* curve and horizontal *x*-axis direction, and b is frequency. This curve was proposed by Hirose after summarizing the body configuration curve during the movement of biological snakes [17].

By using Yamade’ method, we can obtain the angle of each joint when the snake robot adopts the serpentine gait:

(6) $$\left\{\begin{array}{l} \theta_{p}(s)=-2A_{1}L_{0}\cdot \sin(\psi (0))-\frac{2B_{1}}{\omega_{1}}\;\sin(\omega_{1}L_{0})\;\cdot \sin(\psi (0))\;\cdot \sin(\omega_{1}s+\varphi_{1})\\ \theta_{y}(s)=2A_{1}L_{0}\cdot \cos(\psi (0))+\frac{2B_{1}}{\omega_{1}}\sin(\omega_{1}L_{0})\;\cdot \cos(\psi (0))\;\cdot \sin(\omega_{1}s+\varphi_{1}) \end{array}\right.$$

When $A_{1}\neq 0$, the snake robot turns in space with a sinuous gait, and the effect of turning is related to the value of $A_{1}$. When $A_{1}=0$, the snake-shaped robot moved along a straight line. The serpentine motion can be obtained by smoothly changing values of ψ(0) and $\varphi_{1}$ with *t*. Specifically, when ψ(0)=0, the offset of angle are all 0, resulting in the angle of the pitch joint is constant at 0, and the angle of the yaw joint is $\theta_{y,A}\;\sin(\omega_{1}s+\varphi_{1})$, where $\theta_{y,A}=2B_{1}\sin(\omega_{1}L_{0})\;/\omega_{1}$ is a fixed value, and the pitch joint angle is a sine function that changes with *t*.

#### 3.1.2. Rolling Gait (*B*_1_ = 0, *A*_2_ = 0, *B*_2_ = 0)

Rolling gait is another typical parametric gait. When using rolling gait, the snake robot bends into an arc and rolls forward along the body axis, so it is called rolling gait [8]. Let $A_{2}=0$ and $B_{2}=0$, and we can obtain the target configuration curve of the snake robot rolling gait: $\kappa (s)=A_{1}$, τ(s)=0. Likewise, the target configuration curve of snake robot changes from three-dimensional space curve to two-dimensional plane curve because torsion is constant to 0. For rolling gait, the target configuration can only be achieved by changing $A_{1}$.

The angle of each joint can be obtained:

(7) $$\left\{\begin{array}{l} \theta_{p}(s)=-2A_{1}L_{0}\cdot \sin(\psi (0))\\ \theta_{y}(s)=2A_{1}L_{0}\cdot \cos(\psi (0)) \end{array}\right.$$

where $A_{1}$ and $L_{0}$ are fixed value, and different values are smoothly given ψ(0) over time, which can control the snake robot to realize rolling motion.

#### 3.1.3. Helix Rolling Gait (*B*_1_ = 0, *B*_2_ = 0)

Helix rolling gait is another kind of rolling gait, which belongs to a simple three-dimensional space gait. It was first proposed by the Choset team, and it can crawl on rods and pipes through spiral rolling [18,19]. The target configuration curve is a helix, and the curvature and deflection are constant values. Let $B_{1}=0$, $B_{1}=0$, and we obtain the target configuration curve of helix rolling gait: $\kappa (s)=A_{1}$, $\tau (s)=A_{2}$.

Likewise, the angle of each joint can be obtained:

(8) $$\left\{\begin{array}{l} \theta_{p,i}=-2\frac{A_{1}}{A_{2}}\sin(A_{2}L_{0})\cdot \sin(A_{2}s+\psi (0))\\ \theta_{y,i}=2\frac{A_{1}}{A_{2}}\sin(A_{2}L_{0})\cdot \cos(A_{2}s+\psi (0)) \end{array}\right.$$

where $A_{1}$, $A_{2}$ and $L_{0}$ are fixed value. We can control the snake robot to realize rolling motion by changing the value of ψ(0) with time.

### 3.2. Example Gait for Scripted Gaits

The most widely used method in scripted gaits is based on simple curve connecting, that is, connecting straight lines and arcs into the backbone curve of snake robots and extracting control function [14]. Taking crawler gait as an example, this paper shows how the MCC method can realize the control function for the consistency of scripted gait. Other scripted gaits can be achieved through a similar process.

**Figure 3:**
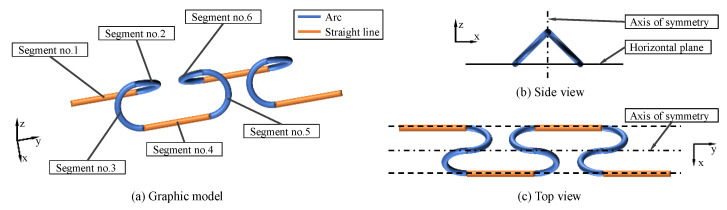
Target configuration curve of crawler gait. The curve is composed of two straight lines and four arcs. When the snake robot moves with crawler gait, the side view of the configuration curve is an isosceles triangle.

Based on the method in [14], the crawler gait was proposed [14]. The backbone curve of this gait is shown in the Figure 3. Although the method of connecting simple curve approximating continuous smooth curve meets the control requirements of crawler gait to a great extent, its motion control function is complex, and the angular and angular acceleration will suddenly change at connection points.

By extracting the main characteristics of the backbone curve of the crawler gait, we can obtain a new simplified crawler gait, which is named the S-crawler gait. The configuration curve of the S-crawler gait only contains the basic configuration information of the crawler gait, and there is no sudden change in the curvature and torsion functions. Since there is no connecting segment, the torsion compensation angle is eliminated. Therefore, the joint angles obtained are continuously differentiable. The process of the ideal configuration curve of the S-crawler gait is described in detail below.

The crawler gait is composed of two straight lines and four arcs, as shows in Figure 3. The straight line segment is in direct contact with the ground to improve the stability of the robot motion and avoid the collision of the arc parts on both sides of the straight line segment due to too small distance; the arc segment is used to form a spatial configuration and generate the spatial motion of the robot. Since the crawler gait configuration curve is composed of six segment curves, it is difficult to directly analyze the simple target configuration curve. Therefore, the crawler gait configuration curve needs to be simplified.

**Figure 4:**
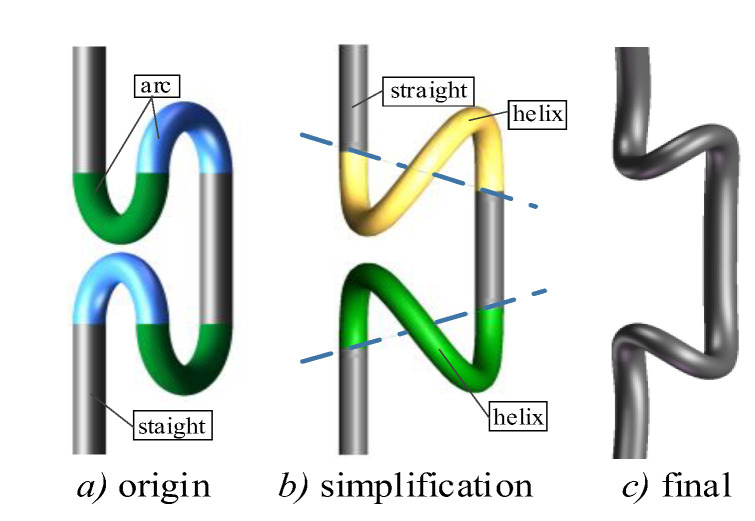
S-crawler gait generation process.

The gait transformation process Is divided into two steps in Figure 4. First, the adjacent arc part is replaced by helix, and the gait target configuration curve changes from six segments to four. The purpose of replacement is simplified from the model. The simplified process is shown in Figure 4a,b. Then, based on the main characteristic curve equation, the simplified target configuration curve is fitted to obtain the analytical equation of the S-crawler gait target configuration curve.

The S-crawler gait configuration curve does not have a straight line segment in the true sense. However, when the snake-shaped robot moves with the S-crawler gait, under the influence of external factors such as gravity, the part in contact with the ground can produce an approximate straight line effect. The curvature of the S-crawler gait configuration curve is gradual, rather than abrupt, and the straight line segment is not necessary. Therefore, when the length of the approximate straight line is 0, the S-crawler gait can still be achieved by changing the rate of curvature gradient. The curvature and torsion parameters of the S-crawler gait target configuration curve are analyzed below.

#### 3.2.1. Curvature *κ*(*s*)

Assuming that the length of a single cycle of the crawler gait configuration curve is L, the ratio of the length of the approximate straight line to the total length is λ(0≤λ<1). After fitting the crawler gait configuration curve based on MCC, in one cycle, the curvature of the S-crawler gait configuration curve has two maxima and two minima, so according to the characteristics of the sine function, it can be obtained:

(9) $$\omega_{1}=4\pi /L$$

(10) $$\sin(\omega_{1}(\lambda L/4)+\varphi_{1})=-1$$

Since the sine curve cannot be a constant value in the segment, if the S-Crawler target configuration curve can obtain an effect similar to the Crawler gait configuration, we set the integral of the S-Crawler gait configuration curve at 0 ≤ *s* < *λL*/2, *L*/2 ≤ *s* < (1 + *λ*)*L* to be 0, form an approximate straight line; the integral of the S-Crawler gait configuration curve at *λL* < *s* < *L*/2, (1+*λ*)*L*/2 < *s* < *L* to be 2*π*, form a bulge. So:

(11) $$\int_{0}^{\lambda L/2}\kappa (s)ds=0\;\mathrm{or}\;\int_{L/2}^{(1+\lambda )L/2}\kappa (s)ds=0$$

(12) $$\int_{\lambda L/2}^{L/2}\kappa (s)ds=2\pi \;\mathrm{or}\;\int_{(1+\lambda )L/2}^{L}\kappa (s)ds=2\pi$$

Now, we obtain the curvature equation of the S-crawler gait configuration curve:

(13) $$\kappa (s)=\frac{4\pi }{L}+B_{1}\cdot \sin(\frac{4\pi }{L}s-\frac{\pi }{2}-\lambda \pi )$$

where $B_{1}$ is equal to any real number if *λ* = 0 or is $\frac{4\pi }{L}\cdot \frac{\lambda \pi }{\sin(\lambda \pi )}$ if 0<λ<1.

#### 3.2.2. Torsion *τ*(*s*)

Using the same method as above, the torsion equation of the S-crawler gait configuration curve can be obtained. In a cycle, the torsion of the S-crawler gait configuration curve has only one maximum value and one minimum value. Therefore, according to the characteristics of the sine function, the following holds:

(14) $$\omega_{2}=2\pi /L$$

(15) $$\sin[\omega_{2}\cdot (\lambda L/2+(1-\lambda )L/4)+\varphi_{2}]=1$$

Let the torsion integral value of the S-crawler target configuration curve in one cycle be 0, and we obtain:

(16) $$\int_{0}^{L}\tau (s)ds=0$$

Now, we obtain the torsion equation of the S-crawler gait configuration curve:

(17) $$\tau (s)=B_{2}\cdot \sin(2\pi s/L-\lambda \pi /2)$$

In order to further simplify the design process, let *λ* = 0, and we can obtain the target configuration curve of the S-crawler gait:

(18) $$\left\{\begin{array}{l} \kappa (s)=\frac{4\pi }{L}+B_{1}\cdot \sin(\frac{4\pi }{L}s-\frac{\pi }{2})\\ \tau (s)=B_{2}\cdot \sin(\frac{2\pi }{L}s) \end{array}\right.$$

**Figure 5:**
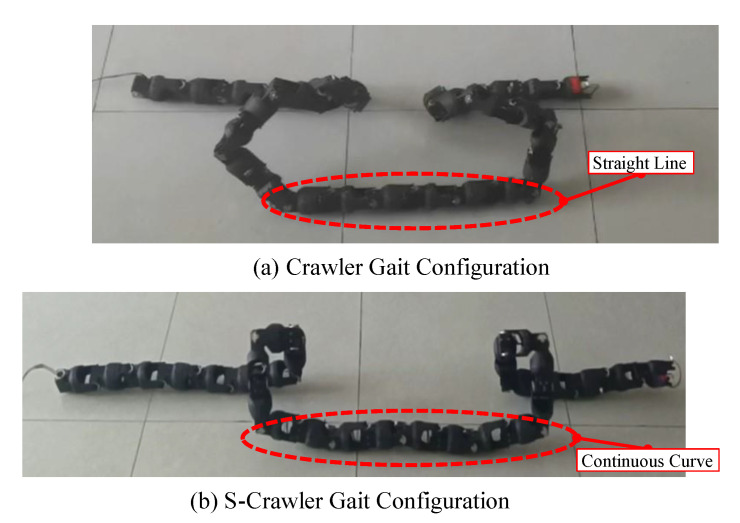
Realization of snake robot with s-crawler gait. The crawler gait configuration and S-crawler gait configuration of the serpentine robot are shown in (**a**,**b**), respectively. The most obvious difference between them is that the straight segment of the original configuration changes into a continuous curve segment.

The S-Crawler gait is proposed by abstracting the target configuration curve of the crawler gait, and only the main features are retained. Therefore, it is necessary to verify whether the S-crawler gait designed based on the main characteristic curve also has the configuration of the crawler gait. In (18), $L=26L_{0}$($L_{0}$ is the length of snake link), $B_{1}=4.8\pi /L$, $B_{2}=2.5\pi /L$. The Crawler gait configuration and the S-crawler gait configuration are, respectively, realized with a serpentine robot in Figure 5. The obvious result is that there is no straight line in the S-crawler gait configuration compared to the crawler gait. In addition, since the configuration curve of S-crawler gait is continuous, the obtained joint angle is also continuous in time domain, and there is no sudden change in angular velocity and acceleration.

## 4. The AEH-Sidewinding Gait Generation

The side-winding gait is a kind of gait that snake robots often adopt when passing through soft ground. The kinematics modeling of traditional side-winding gait is based on the assumption that its backbone curve is an elliptical helix. However, the backbone curve of side-winding motion generated by the parameterized gait method is quite different from the elliptical helix curve. We generate a new AEH-sidewinding gait based on the MCC method, and its backbone curve is close to the ideal elliptic helix, so it is helpful to improve the accuracy of the kinematics model of side-winding gait.

**Figure 6:**
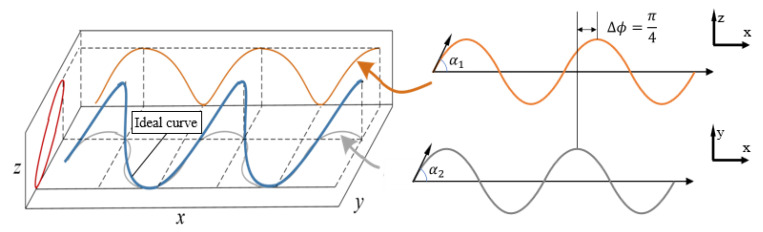
Target Configuration Curve of Sidewinding Gait. The target configuration curve can be seen as the spatial superposition of serpenoid curves in the horizontal and vertical directions, and the phase difference between them is π/4.

In (5), when δ=π/4, we can obtain the angle of each joint when the snake robot moves with sidewinding gait, and its ideal configuration curve is shown in Figure 6 The projection on the XOY and YOZ planes is a serpenoid curve, and the projection on the XOY plane is a ring. Therefore, when analyzing the sidewinding gait motion, the approximately continuous backbone curve of the snake robot is often simplified to an elliptical spiral curve [20]. However, when the length of the joint link is infinitely close to 0, the continuous backbone curve of the snake robot obtained by using the parametric equation is not the same as the ideal curve.

**Figure 7:**
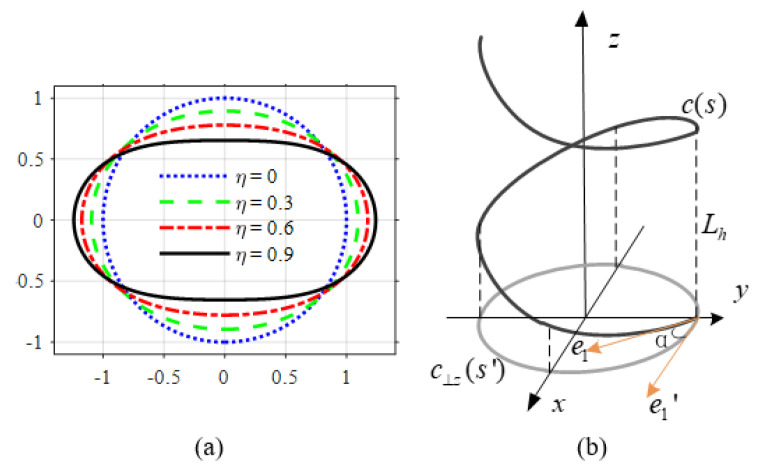
(**a**) Approximate Elliptic Curve. (**b**) Approximate Elliptic Helix Curve.

Therefore, in order to simplify the analysis model, a new type of sidewinding gait target configuration curve is proposed based on the proposed main characteristic curve, as shown in Figure 7.

First, the solution of the curvature equation of the elliptic helix is complicated. In order to simplify the calculation, we propose an approximate plane elliptic curve, as shown in Figure 7a. Its curve equation is:

(19) $$\kappa^{\prime }(s^{\prime })=\frac{2\pi }{L^{\prime }}(1+\eta \cdot \sin(\frac{4\pi }{L^{\prime }}s^{\prime }+\varphi_{1})),\;\tau (s)=0$$

where $L^{\prime }$ is the perimeter of the approximate ellipse line, the integral of $\kappa^{\prime }(s^{\prime })$ from 0 to $L^{\prime }$ is 2π. η(0≤η≤1) is the adjustment coefficient, and, through the changed value, the ellipse line of different shapes can be obtained, and, especially when η = 0, the curve is a circular arc line.

By integrating the curvature $\kappa^{\prime }(s^{\prime })$, we can obtain the angle $\phi (s^{\prime })$ between any point $s^{\prime }$ on the curve and the *x*-axis. The increments in the *x* and *y* directions can be obtained:

(20) $$\phi (s^{\prime })=\int_{0}^{s^{\prime }}\kappa^{\prime }({\hat{s}}^{\prime })d{\hat{s}}^{\prime }+\phi (0)$$

(21) $$dx=\cos(\phi (s^{\prime }))ds^{\prime },\;dy=\sin(\phi (s^{\prime }))ds^{\prime }$$

Now, the analysis of the points on the approximate elliptic curve has been obtained. However, this curve is only a two-dimensional space curve, which cannot meet the design requirements of three-dimensional gait. Therefore, some twist must be applied to the curve, as shown in Figure 7b.

Assuming that the geometric axis of the approximate elliptical helix *c*(*s*) is collinear with the *z*-axis of the coordinate system, the projection of c(s) on the plane oxy is an approximate ellipse $c_{⊥z}(s^{\prime })$, where $dz/ds^{\prime }=a(a\geq 0)$. Because the increment in the *z*-axis direction is a constant value, then $ds=||{\left[dx,dy,dz\right]}^{T}||$$=\sqrt{1+a^{2}}ds^{\prime }$, so:

(22) $$L_{s^{\prime }}=1/\sqrt{a^{2}+1}L_{s},\;L_{h}=a/\sqrt{a^{2}+1}L_{s}$$

where $L_{s}$, $L^{\prime }$ are, respectively, length of the configuration curve and the length of projection on the horizontal plane in a single period, and $L_{h}$ is the pitch.

Based on the Frenet-Serret equation with curvature k(s) and torsion τ(s) to express the curve of approximate elliptical helix, the following holds:

(23) $$\left\{\begin{array}{l} dc(s)/ds=e_{1}(s)\\ de_{1}(s)/ds=\kappa (s)e_{1}(s)\\ de_{2}(s)/ds=-\kappa (s)e_{1}(s)+\tau (s)e_{3}(s)\\ de_{3}(s)/ds=-\tau (s)e_{2}(s) \end{array}\right.$$

Combined with (19), the configuration curve of the approximate elliptical helical sidewinding (AEH-sidewinding) gait can be obtained:

(24) $$\left\{\begin{array}{l} \kappa (s)=\frac{1}{\sqrt{a^{2}+1}}\frac{2\pi }{L_{s}}(1+\eta \cdot \sin(\frac{4\pi }{L_{s}}s+\varphi_{1}))\\ \tau (s)=\frac{a}{\sqrt{a^{2}+1}}\frac{2\pi }{L_{s}}(1+\eta \cdot \sin(\frac{4\pi }{L_{s}}s+\varphi_{1})) \end{array}\right.$$

By changing a, η and $L_{s}$, different forms of elliptical helix can be obtained. In particular, when a = 0, η = 0, a plane arc curve can be obtained; when a = 0, η ≠ 0, a typical cylindrical helix can be obtained; when a→∞, a straight line can be obtained.

**Figure 8:**
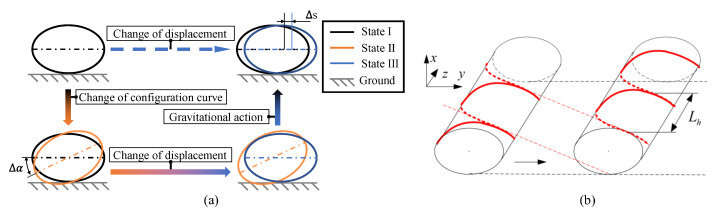
AEH-sidewiding gait movement principle. (**a**) Schematic diagram of movement principle. The state I represents a stable state in equilibrium under the action of gravity, and state II shows the shape after configuration change, and state III is the stable state after the configuration changes and moves under the action of gravity. The space movement of the snake robot is realized by changing the configuration state in a cycle. (**b**) Schematic diagram of motion results of snake robot adopting AEH-sidewinding gait.

When the snake robot adopts AEH-sidewinding gait (movement direction is parallel to the *x*-axis), as shown in Figure 8a, the projection of this curve on the XOY plane is always approximately elliptical. In the initial state, the robot is in a stable state (I), and when the position of the snake robot head on the target configuration curve is changed, the snake robot is in an unstable state (II). Under the influence of friction and gravity, the approximate ellipse is subjected to a torque that causes itself to rotate until it reaches a new stable position (III). By changing the position of the snake robot head on the target configuration curve over time, a continuous rolling motion can be obtained, as shown in Figure 8b.

**Figure 9:**
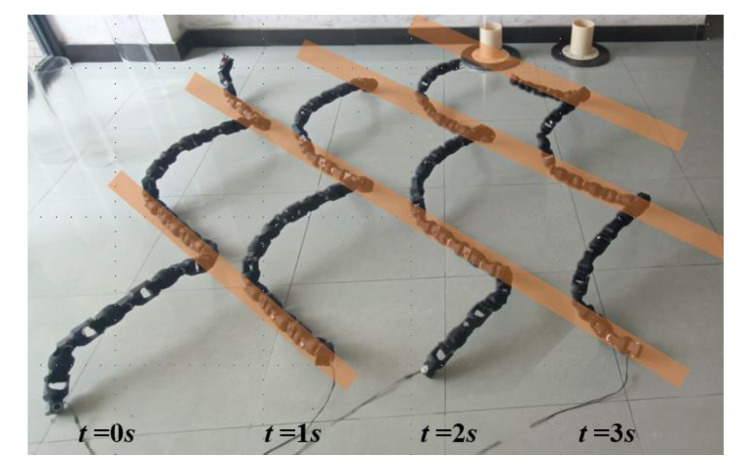
Implementation of snake robot with AEH-sidewinding gait. The figure shows the motion state of a snake robot at t = 0 s, t = 1 s, t = 2 s, and t = 3 s, respectively.

The effectiveness of the AEH-sidewinding gait is verified by snake robot experiment. Let a = 1; η = 0.95; $L_{s}$ = 14$L_{0}$, and obtain the control functions of the AEH-sidewinding gait based on (24). The motion result of the snake robot is shown in Figure 9. The adopted snake robot has a total of 32 joints, which are alternately distributed by pitch joints and yaw joints.

## 5. Conclusions

In this paper, a new gait generation method of snake robots, the MCC method, is proposed. By abstracting the low-order sine factor of the backbone curve of snake robots, the unified control expression function of various gaits is obtained. Serpentine gait, rolling gait, helix rolling gait based on parameterized gait generation method, and crawler gait based on scripted gait generation method are reconstructed by the MCC method. In addition, for the mismatch between the traditional side-winding gait and the kinematics modeling of the side-winding gait based on the assumption of ideal elliptic helix, an AEH-sidewinding gait with a back curve closer to the ideal elliptic helix is proposed and verified by experiments. This unified gait expression of snake robot will be helpful to realize smooth gait switching between different gaits of snake robot.

## Data Availability

Not applicable.
